# Rapid Onsite Visual Detection of Orf Virus Using a Recombinase-Aided Amplification Assay

**DOI:** 10.3390/life13020494

**Published:** 2023-02-10

**Authors:** Huan Cui, Jiyu Guan, Huijun Lu, Jun Liu, Fei Tu, Cheng Zhang, Kai Su, Zhendong Guo, Kui Zhao

**Affiliations:** 1 College of Veterinary Medicine, Jilin University, Changchun 130062, China; 2 Key Laboratory of Zoonosis, Ministry of Education, Institute of Zoonosis, Jilin University, Changchun 130062, China; 3 Changchun Veterinary Research Institute, Chinese Academy of Agriculture Sciences, Changchun 130122, China; 4 College of Veterinary Medicine, Hebei Agricultural University, 2596 Lucky South Street, Baoding 071000, China

**Keywords:** recombinase-aided amplification (RAA), ORFV, isothermal amplification, rapid diagnosis, DNA extraction-free

## Abstract

Orf is an important zoonotic disease caused by the Orf virus (ORFV) which can cause contagious pustular dermatitis in goats and sheep. Orf is widespread in most sheep-raising countries in the world, causing huge economic losses. Although diagnostic methods for ORFV infection already exist, it is still necessary to develop a time-saving, labor-saving, specific, low-cost and visual diagnostic method for rapid detection of ORFV in the field and application in grassroots laboratories. This study establishes a DNA extraction–free, real-time, visual recombinase–aided amplification (RAA) method for the rapid detection of ORFV. This method is specific to ORFV and does not cross-react with other common DNA viruses. The detection limits of the real-time RAA and visual judgment of the RAA assay at 95% probability were 13 and 21 copies per reaction for ORFV, respectively. Compared with qPCR, the sensitivity and specificity of the real-time RAA assay were 100%, and those of the visual RAA assay were 92.31% and 100.0%, respectively. The DNA extraction–free visual detection method of RAA established in this study can meet the needs of rapid onsite detection and grassroots laboratories and has important reference value and significance for the early diagnosis of diseased animals.

## 1. Introduction

Poxviridae is a widely known virus family with the largest number of members of any virus; it is the largest and most complex virus known to harm mammals. It infests mammals, including humans. Recently, increasing attention has been given to it [1,2]. The origin and evolution of poxviruses remain obscure, despite evidence that poxviruses emerged thousands of years ago. Poxvirus genomes evolved through the acquisition and loss of genes, especially gene replication and horizontal gene transfer, to adapt to and combat the host’s antiviral immune defenses to establish successful infection [3]. Parapoxvirus belongs to Chordopoxvirinae, a vertebrate Poxviridae, and is one of the most commonly studied genera of poxvirus. The members of parapoxvirus include Orf virus (ORFV), bovine papular stomatitis virus (BPSV), pseudocowpox virus (PCPV), and the newly discovered parapoxvirus of red deer in New Zealand (PVNZ) [4,5]. Compared with orthopoxvirus, paraxxvirus is relatively small in size and has a high content of G + C in the genome [6,7].

ORFV is an epitheliophilic double-stranded DNA virus. It belongs to the genus parapoxvirus of the family Poxviridae. ORFV can cause infectious pustulosis in goats and sheep and is widespread in most sheep-raising countries in the world [8]. Lambs aged 1–6 months are most susceptible to ORFV infection [9]. Symptoms such as muzzle skin or mucous membrane ulcers, papules, abscesses, scabs, and other symptoms are characteristic of ORFV infection in sheep [9]. Infected sheep experience decreased feed intake, slow growth and development, and rapid spread throughout the breeding area, causing serious economic losses to the farm [10]. Orf outbreaks have occurred in many countries, causing huge economic losses to the breeding industry [11,12,13,14,15]. Orf is a zoonotic disease, and farm workers and others who have direct or indirect contact with ORFV-infected animals can occasionally become infected with ORFV through skin wounds [10]. ORFV can be routinely diagnosed on the basis of typical skin lesions, clinical signs, and epidemic symptoms. However, the clinical symptoms of infection with ORFV can be remarkably similar to those of other viruses (such as sheep pox virus and goat pox virus), making differential diagnosis difficult. Therefore, it is often necessary to rely on laboratory diagnosis. Currently, electron microscopy and pathogenic detection (polymerase chain reaction and real-time quantitative PCR) are the primary laboratory diagnostic methods for ORFV, which rely on expensive equipment or professional operation [16]. Therefore, the development of a time-saving, labor-saving, specific, visual, and low-cost diagnostic method is crucial for the clinical diagnosis of ORFV.

Polymerase chain reaction (PCR), which was invented in the 1980s [17], has been widely used in food safety, molecular biology, medicine, and other fields as a technique for amplifying specific DNA fragments [18,19,20]. PCR can be used to detect pathogenic microorganisms with high sensitivity and specificity. In particular, precise quantification of target detection can be achieved through digital PCR [21,22,23]. However, digital PCR relies on precision instruments for temperature control and has the defect of an overly long reaction time, which makes the technology restricted in field detection and popularization at the grassroots level [24]. Quantitative real-time PCR (qRT-PCR) is the most commonly used method to detect pathogens of infectious diseases [25,26]. However, such tests require tedious sample pretreatment, nucleic acid extraction and purification, and complex thermal cycling processes, and rely on expensive testing equipment and specialized laboratory conditions. Therefore, qRT-PCR cannot meet the requirements of real-time detection in the field. In the early 1990s, some scholars began to try nucleic acid isothermal amplification technology, combining it with other technologies to the extent that nucleic acid isothermal amplification technology is widely used in many fields [27]. Compared with PCR and qRT-PCR, nucleic acid isothermal amplification technology does not require too long a reaction time, nor does it have overly harsh requirements with regard to reaction instruments; moreover, it can realize rapid screening and detection of pathogenic microorganisms [28].

Due to the characteristics of nucleic acid amplification at specific temperatures, isothermal amplification technology has attracted wide attention. The specific technologies include strand displacement amplification (SDA), rolling circle amplification (RCA), loop-mediated isothermal amplification (LAMP), helicase-dependent isothermal DNA amplification (HDA), recombinase polymerase amplification (RPA), and recombinase-aided amplification (RAA). In 1992, Walker et al. discovered SDA, a novel DNA amplification technique [29]. SDA technology is based on enzymatic reactions to perform in vitro isothermal amplification of DNA. The whole SDA reaction basically relies on restriction endonuclease to cut the complementary chain of semithiophosphorylated base pairs and polymerase exo-Klenow to extend the incision and replace downstream DNA fragments to achieve efficient amplification of target sequences. In 1995, Fire et al. first proposed RCA, a technique that simulates the rolling ring replication mode of circular molecules in nature [30]. A primer that is complementary to the target sequence is along the circular DNA template. The length of single-strand DNA amplified by chain replacement by DNA polymerase with chain replacement activity can be increased by nearly one thousand times. RCA is widely used in gene chips, nucleic acid sequencing, etc. The specific generation of circular DNA is a key part of this technology. Notomi et al. studied LAMP in 2000 [31]. LAMP can design four specific primers on the basis of six regions in the target genes and then complete the amplification with the help of strand replacement DNA polymerase (Bst DNA polymerase) in 10 min at a constant temperature of approximately 65 °C. This innovation not only omits the conditions of thermal degeneration of the template and maintains a permanent temperature cycle but also has the characteristics of high specificity, high efficiency, short time, low cost, and easy operation. In 2004, HDA emerged [32]. Here the reaction principle mimics the replication of DNA in vivo under isothermal conditions. The DNA double strand relies on helicase for unwinding at constant temperature. The specific primer takes the unwrapped single strand stabilized by the DNA single-strand binding protein as the template and relies on DNA polymerase to synthesize a complementary double strand for recirculation amplification. In 2006, Piepenburg et al. initially proposed RPA technology involving two specific upstream and downstream primers, recombinase enzyme (T4 uvsX), polymerase (Bsu), and single-chain binding protein (gp32) [33]. The amplified products of RPA are usually combined with fluorescence probes, agarose gel electrophoresis, side flow chromatography strip detection, and other methods. Thus, nucleic acid isothermal amplification technology has been further developed [34].

Nucleic acid isothermal amplification technology has potential application prospects due to rapid onsite amplification without complex or sophisticated laboratory equipment [35]. Recombinase-aided amplification (RAA) is a new isothermal amplification technology that takes approximately 30 min under isothermal conditions at 37–42 °C [10,36]. The RAA system contains three essential enzymes. A protein known as single-stranded DNA binding protein (SSB) specifically binds to single-stranded DNA regions; recombinases (which pair specific primers with template DNA); and strand displacement DNA polymerization enzymes (for extension and DNA amplification) [37,38]. By adding certain fluorescent probes to the RAA system, DNA amplification products can be monitored in real time. Moreover, combining the CRISPR system with RAA technology enables ultrasensitive and specific detection of DNA or RNA molecules [39,40]. Attention to RAA has been increasing in recent years because of its ability to detect various pathogenic microorganisms. The numerous advantages of this technique include simple primer design, fast amplification speed, high sensitivity, low equipment requirements, no need for expensive equipment, simple operation, and intuitive result evaluation [41,42,43,44,45,46].

The present study established a DNA extraction–free, visual RAA method for the rapid detection of ORFV. The method has high sensitivity, strong specificity, and simple operation. Portable blue light equipment can be used to visually view amplicons, making the method suitable for ORFV detection in emergency and low-resource circumstances.

## 2. Materials and Methods

### 2.1. Virus and Clinical Samples of ORFV

The studied ORFV OV-SY17 strain (GenBank accession number MG712417) was stored in our laboratory [47]. Seventy-eight clinical samples (diseased skin tissues) of suspected ORFV infection were collected from sheep farms in Hebei Province, northern China, in 2021. All the diseased skin tissue samples from the sheep were collected after obtaining informed consent from the owner. Clinical samples were preserved in phosphate-buffered saline (PBS) and stored at −80 °C until use. All the experiments were performed at Changchun Veterinary Research Institute, Chinese Academy of Agriculture Sciences.

### 2.2. Primers and Probe Design for RAA

**Figure 1:**
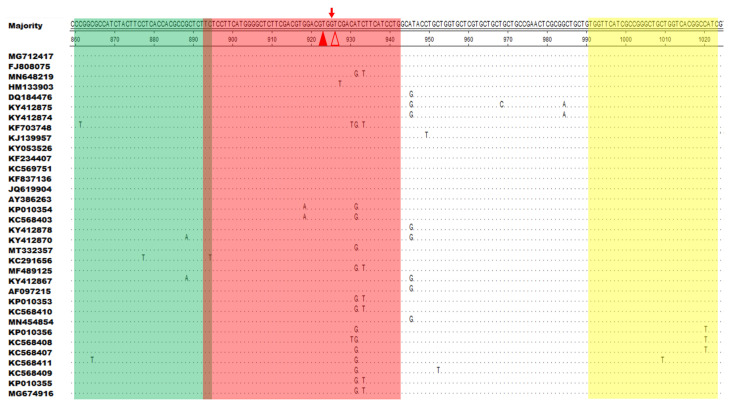
Positions of the primers and probe used for recombinase-aided amplification (RAA) in the F1L gene sequence of different ORFV strains in the GenBank database. Several corresponding nucleotide residues that match the majority are indicated by dots. The forward primer (F860–894) is indicated as green, the reverse primer (R991–1023) as yellow, and the exo probe (p893–942) as red. The two T residues within p893–942 labeled with a fluorophore (FAM) and quencher (BHQ1) are marked with solid and hollow triangles, respectively.

DNASTAR software was used to compare 34 different ORFV F1L gene sequences (downloaded from the GenBank database), and SnapGene software (version 4.3.6) was used to design primers and probes. According to the method reported in our previous study, the optimal primers and probes were selected [48]. In simple terms, a good primer pair can be found by screening all reverse primers with one forward primer (chosen at random), choosing the best reverse primer and using it to screen all forward primers. Furthermore, the primers and probes for the qPCR detection of the ORFV F1L gene were synthesized as described in a previous study (Table 1) [49]. All the primers and probes were synthesized by Comate Biotech Co., Ltd. (Changchun, China). Figure 1 and Table 1 list the RAA amplification primers and probes designed in this study.

### 2.3. Pyrolysis (DNA Extraction–Free) of Clinical Samples

After being removed from PBS, the diseased skin tissues were cut into small pieces (0.2–0.3 g) using ophthalmic scissors. Subsequently, the scissored tissue was placed directly in 100 µL MightyPrep reagent for DNA (TaKaRa, Dalian, China) and heated for 10 min at 95 °C. The supernatant was used as the template for the RAA and qPCR assays.

### 2.4. Real-Time PCR and DNA Extraction–Free RAA Assay

The qPCR experiments were performed using Probe qPCR Mix (TaKaRa, Dalian, China). The qPCR system of ORFV is described as follows: a 25 μL reaction mixture comprising 12.5 μL 2× Probe qPCR Mix, 0.5 μL forward primer (10 μM), 0.5 μL reverse primer (10 μM), 1.0 μL probe (10 μM), 8.25 μL nuclease-free water, 50× ROX Reference Dye II 0.25 μL, and nucleic acid template 2.0 μL. The reaction tubes were placed into the 7500 real-time PCR System (Applied Biosystems) with an initial step at 95 °C for 30 s, followed by 40 cycles at 95 °C for 5 s and 60 °C for 35 s. The RAA assay was performed using a kit (#WLE8202KIT) manufactured by Amp-Future Biotech Co., Ltd. (Weifang, China). Briefly, the RAA system (25 µL per reaction) contained 14.7 µL of buffer A, 1.25 μL of buffer B, 1.0 µL of forward primer (10 µM), 1.0 µL of reverse primer (10 µM), 0.3 µL of exo probe (10 µM), 4.75 μL of RNase-free H_2_O, and 2.0 μL of the template. The tubes were placed in a 7500 real-time PCR system (Applied Biosystems), the temperature was adjusted to 42 °C, and the reaction was continued for 30 min (one cycle per minute). Using a portable blue light meter (TGreen, Tiangen, Beijing, China) with an excitation wavelength of 480 nm, the amplification products were visualized. Three different people were used to judge the visual results at the same time.

### 2.5. Analytical Specificity

Reference was made to published studies; four common and important DNA viruses were used to evaluate the specificity of the RAA assay [10,50]. The five viruses are goat pox virus, sheep pox virus, African swine fever virus, bovine papular stomatitis virus and pseudorabies virus. African swine fever virus is stored in Biosafety Laboratory Level 3, Changchun Veterinary Research Institute, Chinese Academy of Agricultural Sciences. Viral RNA of the four viruses was extracted as a template to evaluate the specificity of the RAA assay.

### 2.6. Analytical Sensitivity

The ORFV-F1L plasmid (pMD18-T-F1L, which was kept in our laboratory) was diluted by a 10-fold ratio to reach concentrations of 1 × 10^5^ to 1 × 10^−1^ copies per 2 µL. Two microliters of each diluted plasmid was used as a template to evaluate the RAA sensitivity. The same template was analyzed separately using the ORFV qPCR assay for comparison. For a more accurate analysis of the limit of amplification, eight replicates were independently run in both assays using the dilution series (10^5^–10^−1^ copies of ORFV-F1L plasmid per reaction) as templates. IBM’s Statistical Product and Service Solutions (SPSS) software was then used to run a probit regression analysis on the data.

### 2.7. Comparison of DNA Extraction–Free RAA with qPCR Using Clinical Samples

ORFV amplification was performed on 78 clinical samples using the DNA extraction-free RAA method. The details of the clinical samples are provided in Supplementary Table S1. Each sample was from a different sheep or goat. Samples were also evaluated by qPCR for comparison.

### 2.8. Statistical Analysis

To determine the expansion limits, probit regression analysis was performed using IBM’s SPSS software at the 95% probability level. The coincidence rates of DNA extraction-free RAA and qPCR were compared using kappa statistics.

## 3. Results

### 3.1. Positions of the RAA Primers and Probe

DNASTAR software was used to align F1L gene sequences from 34 different ORFV strains. Specific primers and probes were designed in the most conserved region of the F1L gene using SnapGene software. Two modified thymine (T) residues in the chosen probe (p893–942) were fully conserved among 34 representative ORFV strains (Figure 1). The best primer pair, F860–894/R991–1023, was obtained after secondary primer screening. Nucleotide residues corresponding to the majority are indicated by dots. The forward primer (F860–894) is green, the reverse primer (R991–1023) is yellow, and the exo probe (p893–942) is red. A fluorophore (FAM) and a quencher (BHQ1) are used to identify the two T residues of p893–942 as solid and hollow triangles, respectively (Figure 1).

### 3.2. Screening the Optimal Primers for RAA Amplification

**Figure 2:**
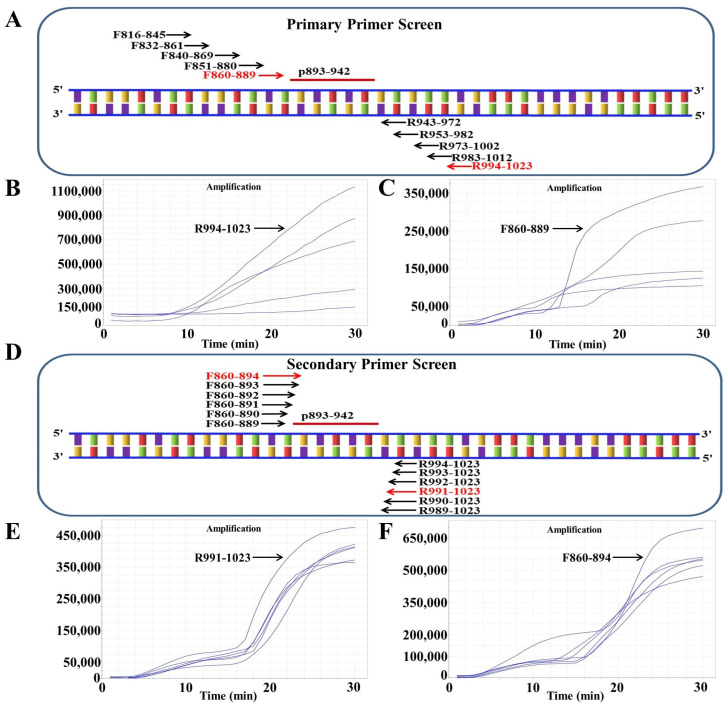
Screening the optical primers for RAA detection. (**A**) Primary primer screening diagram. The positions inside the F1L gene from ORFV are denoted by the numbers in the primer name. (**B**) Reverse primers were used for primary screening. The forward primer F816–845 was randomly selected to screen all five reverse primers. (**C**) RAA primary forward primer screening results. The selected reverse primer R994–1023 was used to screen all five forward primers. (**D**) Sketch map of secondary primer screening. (**E**) Secondary reverse primer screening results. The selected forward primer F860–889 was applied to screen all six reverse primers. (**F**) Secondary forward primer screening results. The selected reverse primer R991–1023 was used to screen all six forward primers.

As described above, we first selected an ideal probe (p893–942) (Figure 2, Table 1). Then, five forward (F816–845, F832–861, F840–869, F851–880, F860–889) and candidate primers were designed around p893–942 (R943–972, R953–982, R973–1002, R983–1012, R994–1023) (Figure 2A). On the basis of previously established methods, the primer was screened [51]. Briefly, five reverse primers were screened with the forward primer F816–845, of which the reverse primer R994–1023 had the best amplification effect (Figure 2B). R994–1023 was applied for experimental screening of all forward primers, and the best effect was observed with F860–889 (Figure 2C). After the initial screening, the primer combination F860–889/R994–1023 was considered to be the best. To screen for better primer combinations, five new forward primers and five new reverse primers around F860–889 and R994–1023 were designed (Figure 2D). Six reverse primers were screened with F860–889, and R991–1023 showed the best amplification (Figure 2E). The six forward primers were then screened using R991–1023; the best results were obtained with F860–894 (Figure 2F). Finally, the optimal primer pair F860–894/R991–1023 was screened out.

### 3.3. Specificity Analysis

**Figure 3:**
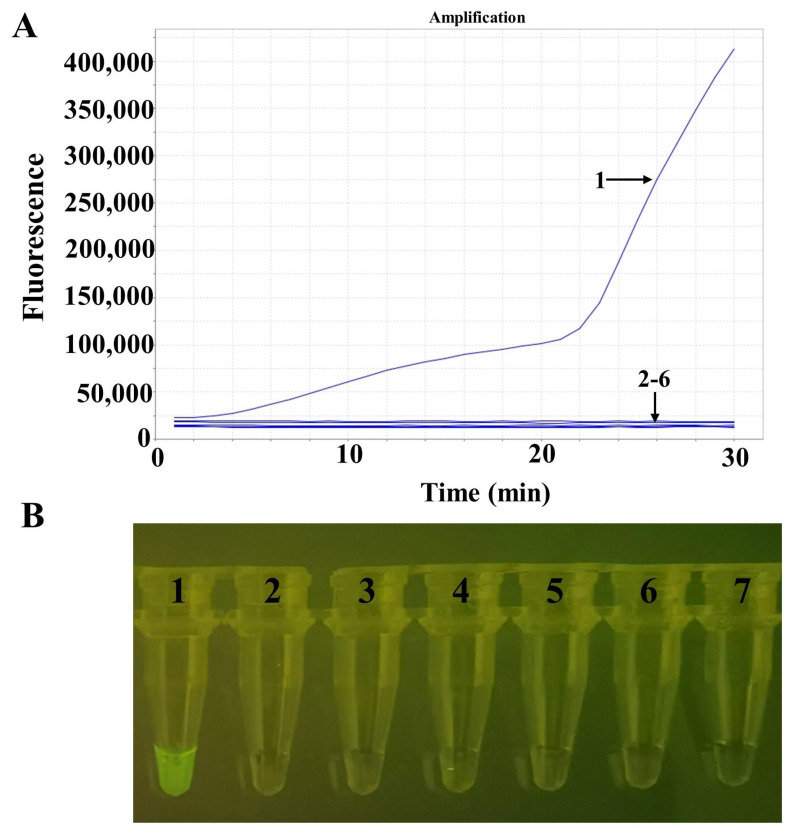
Specificity tests for ORFV. (**A**) The detection results of RAA via real-time fluorescence. (**B**) The detection results of RAA via a portable blue light instrument. Numbers 1–7 indicate nucleic acid templates of ORFV, goat pox virus, sheep pox virus, African swine fever virus, bovine papular stomatitis virus, pseudorabies virus, and the negative control, respectively.

Specificity analysis showed that the RAA assay was positive for ORFV and negative for the goat pox virus, the sheep pox virus, the African swine fever virus, the bovine papular stomatitis virus, the pseudorabies virus, and negative groups (Figure 3A). Figure 3B shows the amplified product visualization of the TGreen instrument.

### 3.4. Sensitivity Analysis

**Figure 4:**
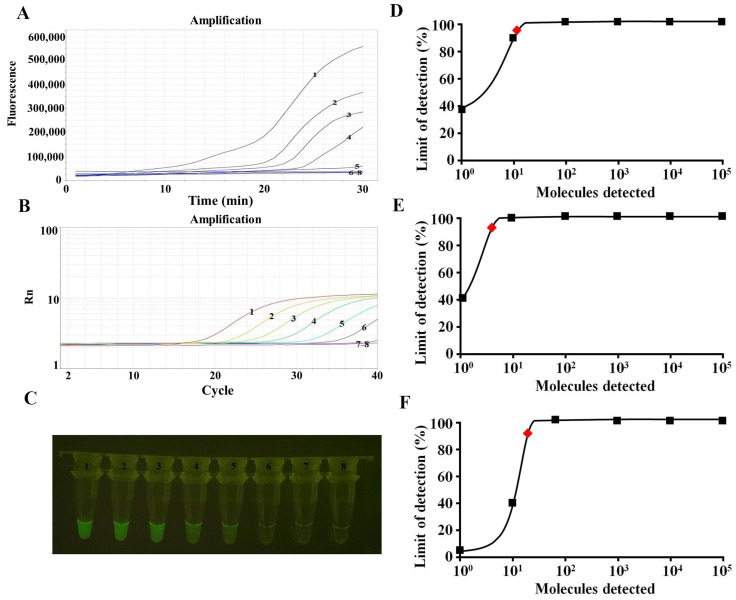
Comparison of sensitivity in detecting ORFV. 1–8 are 10^5^–10^−1^ copies and the negative control, respectively. (**A**) Sensitivity of the RAA assay (real-time fluorescence) for ORFV detection. (**B**) Sensitivity of the qPCR assay for ORFV detection. (**C**) Sensitivity of the RAA assay (visualization) for ORFV detection. (**D**) The results showed that the detection limit of the RAA assay (real-time fluorescence) with 95% reliability per reaction was 13 copies (red rhomboid). (**E**) The results showed that the detection limit of the qPCR assay with 95% reliability per reaction was 4 copies (red rhomboid). (**F**) The results showed that the detection limit of the RAA assay (visualization) with 95% reliability per reaction was 21 copies (red rhomboid).

We performed an RAA assay sensitivity study employing templates made from serially diluted ORFV-F1L plasmid (10^5^–10^−1^ copies per response). The same template was analyzed simultaneously using the qPCR assay for comparison. The amplification limit using real-time fluorescence readout was 10 copies per reaction for the RAA test and 1 copy per qPCR (Figure 4A,B). The sensitivity of the RAA assay for the visualized results was 10 copies per reaction (Figure 4C). Probit regression analyses further showed that for ORFV, the amplification limits of the RAA and qPCR and the visual judgment of the RAA assay were 13, 4, and 21 copies per reaction with 95% probability (Figure 4D–F).

### 3.5. Diagnostic Sensitivity and Specificity of Samples

To evaluate clinical performance, 78 clinical samples were subjected to DNA extraction–free RAA and qPCR amplification. The sensitivity of the DNA extraction–free RAA (through real-time fluorescence readout) assay compared to the qPCR was 100% (39/39) with a specificity of 100% (39/39). The correlation between the two tests was very strong, with a Kappa value of 1 (*p* < 0.001, Table 2). Both positive and negative predictive values (PPV and NPV) were 100%. In addition, the DNA extraction–free RAA (through visual detection) test had a sensitivity and specificity of 92.31% (36/39) and 100.0% (39/39), respectively. The correlation between the two tests was strong, with a Kappa value of 0.923 (*p* < 0.001, Table 2). The NPV was just 92.86%, and the PPV was 100%.

## 4. Discussion

Orf is one of the most important diseases affecting sheep. Although the lethality of ORFV is limited, infected sheep often have difficulty eating, leading to extreme exhaustion and secondary infections, which has caused huge economic losses to the sheep industry around the world. In a recent report, the detection rate of ORFV in northwest China exceeded 40% [52]. The existing ORFV vaccines are not sufficient to guarantee complete protection of goats and sheep from ORFV infection, and the animals may still be infected even after vaccination [11]. There is no proven effective method for medical treatment, so prevention and early diagnosis are crucial.

The molecular detection method consists of three main steps in the conventional scheme: sample collection, nucleic acid extraction, and testing. The latter two steps play an extremely important role in highly sensitive nucleic acid detection [53], so we established a simple and rapid ORFV detection method for RAA without DNA extraction. Rapid and accurate detection of pathogenic microorganisms is essential for early detection, early reporting, early isolation, and early treatment to effectively control the spread of the epidemic [54]. Although many research teams are trying to develop new, rapid, and inexpensive nucleic acid test kits, our ORFV detection of RAA without a DNA extraction test still shows clear advantages. Compared to PCR and qRT-PCR diagnostic methods that rely on the use of purified nucleic acids, our RAA assay eliminates the need for a conventional nucleic acid separation step and reduces the sample preparation time from 2 h to 5 min [55]. Compared with the previously studied PCR and qRT-PCR methods for the detection of pathogenic microorganisms, our assay has significant advantages. The main advantage of the assay is its rapid and qualitative visual effect without the need for professional instrumentation. Specifically, previous detection methods often involved complex operating procedures and expensive instruments, which are not able to be carried out in many cases. Our method is simple to operate, and the results are visual. Therefore, it can be expanded to focus future research on the development of instant diagnosis of pathogenic microorganisms.

Electron microscopy, ELISA, and qPCR have been used to identify ORFV [56]. The development of these methods is essential for the control, eradication, and prevention of Orf. These methods, however, are time-consuming, costly, and sophisticated and require skilled experts, making them unsuitable for general clinical application. Therefore, it is necessary to create a fast, portable, and accurate ORFV detection technology. Previous studies have shown that both the F1L and B2L genes of ORFV exhibit good stability in detection, and both could serve as diagnostic molecular markers [50,57,58]. The RAA method of ORFV detection using the BL2 gene as the target gene combined with nucleic acids has been reported [10]. However, there has been no report on the ORFV F1L gene for ORFV detection in RAA without DNA extraction. In this study, a DNA extraction–free RAA assay was first applied to the visual detection of the ORFV F1L gene. Previous visual detection techniques for ORFV mainly focused on RPA and LAMP assays [58,59]. Although the RPA assay combined with a lateral flow immunoassay strip can also achieve visualization, it increases the cost of detection, which is not conducive to the promotion and application of the technology [60]. Conventional diagnostic methods, such as qPCR, have many problems, such as complex operation, complex equipment, and time consumption, which cannot meet the needs of early and rapid diagnosis in clinical practice. The RAA technology can be amplified under isothermal (37–42 °C) conditions and can be completed within 30 min. The detection template can be either DNA or RNA. The results can be judged visually, which is suitable for point-of-care testing.

The collected clinical samples were further tested by the DNA extraction–free RAA method, and the results showed that, compared with qPCR, the sensitivity and specificity of the DNA extraction–free RAA (via real-time fluorescence readout) assay were 100%. The specificity and sensitivity of the RAA (via visual detection) assay were 92.31% and 100.0%, respectively. The visualization of DNA extraction–free RAA detection can meet the needs of rapid onsite detection and grassroots laboratories and has important reference value and significance for the early diagnosis of diseased animals [61]. In the RAA test, we also used a water bath to react and found that there was no impact on the test results because qPCR was carried out on a fluorescence quantitative instrument. To ensure that the reaction conditions were consistent, we also used a fluorescence quantitative instrument to carry out the RAA reaction. In addition, in accordance with previous studies, there is a recombinant polymerase amplification (RPA) assay to detect ORFV, but this assay has a sensitivity of 100 copies/reaction, so the DNA extraction–free RAA assay is superior to the RPA assay in terms of sensitivity [59]. There is a limitation that must be acknowledged: cost-benefit analysis was not performed in this study, and it will be further investigated in future studies.

## 5. Conclusions

The DNA extraction–free RAA detection method for ORFV established in this study can achieve rapid molecular detection of ORFV in samples within 30 min at 42 °C. The comparison of the detection results of different samples shows that the DNA extraction–free RAA method is consistent with qPCR, and the amplification products can be visualized. Therefore, as a simple and rapid initial screening method, this method can successfully detect ORFV in samples. The detection system is DNA extraction–free; has strong specificity, high sensitivity, and accurate detection results; and does not require complex instruments and equipment. Moreover, it can be visualized onsite. The visual and rapid detection method of ORFV established in this study is of great significance for Orf prevention and provides technical support for Orf control.

## Figures and Tables

**Table 1:** The primers and probes used in the RAA and qPCR tests for ORFV.

Name	Sequences (5′→3′)	Gene	Amplicon Size	Source
F860–894	CCGGCGCCATCTACTTCCTCACCACGCCGCTCTTC	F1L	164 bp	This study
R991–1023	GATGGCCGTGACCAGCAGCCCGGCGATGAACCA	F1L		This study
P893–942 ^a^	TCTCCTTCATGGGGCTCTTCGACGTGGACG(FAM-dT)G(THF)(BHQ1-dT)CGACGTCTTCATCCTG[C3-spacer]	F1L		This study
F	CAGCAGAGCCGCGTGAA	F1L	70 bp	[49]
R	CATGAACCGCTACAACACCTTCT	F1L		[49]
Probe ^b^	FAM-CACCTTCGGCTCCAC-MGB	F1L		[49]

^a^ FAM-dT, thymidine nucleotide carrying fluorescein; BHQ1-dT, thymidine nucleotide carrying black hole quencher 1; THF: tetrahydrofuran spacer; C3-Spacer, C3 spacer at the 3′ end to block elongation. ^b^ FAM, 6-carboxyfluorescein; MGB, minor groove binder.

**Table 2:** Comparison of ORFV DNA extraction–free RAA with qPCR assay on clinical samples.

Assay		qPCR		Sensitivity	Specificity	Kappa
		Positive	Negative			
RAA (Via real-time fluorescence read-out)	Positive	39	0	100%(39/39)	100%(39/39)	1
	Negative	0	39			
RAA (Via visual detection)	Positive	36	0	92.31%(36/39)	100%(39/39)	0.923
	Negative	3	39			

## Data Availability

The study’s original contributions are included in the article; further inquiries can be directed to the corresponding authors.

## Supplementary Materials

The following supporting information can be downloaded at: www.mdpi.com/article/10.3390/life13020494/s1, Table S1: Clinical details for skin tissues samples.
