# Supplementary material for: Rapid Onsite Visual Detection of Orf Virus Using a Recombinase-Aided Amplification Assay
Source: Life (Basel). 2023 Feb 10;13(2):494. doi: 10.3390/life13020494 (PMC9968157; doi:10.3390/life13020494)
Supplement: Supplementary file 1 [file life-13-00494-s001.zip › life-2098027-supplementary.pdf]

Supplementary Table S1. Clinical details for skin tissues samples.

Serial number	Sample condition	Time	Host [a]	Location
1	Positive	2021.01.25	sheep	China: Hebei, Baoding
2	Positive	2021.01.25	sheep	China: Hebei, Baoding
3	Positive	2021.01.25	sheep	China: Hebei, Baoding
4	Positive	2021.01.25	goat	China: Hebei, Baoding
5	Positive	2021.01.25	goat	China: Hebei, Baoding
6	Positive	2021.01.26	sheep	China: Hebei, Cangzhou
7	Positive	2021.01.26	sheep	China: Hebei, Cangzhou
8	Positive	2021.01.28	goat	China: Hebei, Xingtai
9	Positive	2021.01.28	goat	China: Hebei, Xingtai
10	Positive	2021.01.29	sheep	China: Hebei, Shijiazhuang
11	Positive	2021.01.29	sheep	China: Hebei, Shijiazhuang
12	Positive	2021.01.29	goat	China: Hebei, Shijiazhuang
13	Positive	2021.01.29	goat	China: Hebei, Shijiazhuang
14	Positive	2021.02.15	sheep	China: Hebei, Shijiazhuang
15	Positive	2021.02.15	sheep	China: Hebei, Shijiazhuang
16	Positive	2021.02.15	sheep	China: Hebei, Shijiazhuang
17	Positive	2021.02.15	sheep	China: Hebei, Shijiazhuang
18	Positive	2021.02.18	goat	China: Hebei, Baoding
19	Positive	2021.02.18	goat	China: Hebei, Baoding
20	Positive	2021.02.18	goat	China: Hebei, Baoding

21	Positive	2021.02.18	goat	China: Hebei, Baoding
22	Positive	2021.02.18	goat	China: Hebei, Baoding
23	Positive	2021.02.18	goat	China: Hebei, Baoding
24	Positive	2021.02.22	sheep	China: Hebei, Dingzhou
25	Positive	2021.02.24	goat	China: Hebei, Baoding
26	Positive	2021.02.24	goat	China: Hebei, Baoding
27	Positive	2021.02.24	goat	China: Hebei, Baoding
28	Positive	2021.02.25	sheep	China: Hebei, Dingzhou
29	Positive	2021.02.26	goat	China: Hebei, Handan
30	Positive	2021.02.26	goat	China: Hebei, Handan
31	Positive	2021.02.26	sheep	China: Hebei, Handan
32	Positive	2021.02.26	goat	China: Hebei, Handan
33	Positive	2021.02.26	goat	China: Hebei, Handan
34	Positive	2021.02.26	goat	China: Hebei, Handan
35	Positive	2021.03.12	sheep	China: Hebei, Baoding
36	Positive	2021.03.12	goat	China: Hebei, Baoding
37	Positive	2021.03.12	goat	China: Hebei, Baoding
38	Positive	2021.03.15	sheep	China: Hebei, Baoding
39	Positive	2021.03.15	sheep	China: Hebei, Baoding
40	Negative	2021.01.28	goat	China: Hebei, Zhangjiakou
41	Negative	2021.01.28	sheep	China: Hebei, Zhangjiakou
42	Negative	2021.01.28	sheep	China: Hebei, Zhangjiakou

43	Negative	2021.02.03	goat	China: Hebei, Qinhuangdao
44	Negative	2021.02.03	goat	China: Hebei, Qinhuangdao
45	Negative	2021.02.03	sheep	China: Hebei, Qinhuangdao
46	Negative	2021.02.03	goat	China: Hebei, Qinhuangdao
47	Negative	2021.02.12	sheep	China: Hebei, Baoding
48	Negative	2021.02.12	sheep	China: Hebei, Baoding
49	Negative	2021.02.15	goat	China: Hebei, Baoding
50	Negative	2021.02.15	goat	China: Hebei, Baoding
51	Negative	2021.02.17	sheep	China: Hebei, Zhangjiakou
52	Negative	2021.02.17	goat	China: Hebei, Zhangjiakou
53	Negative	2021.02.17	goat	China: Hebei, Zhangjiakou
54	Negative	2021.02.17	sheep	China: Hebei, Zhangjiakou
55	Negative	2021.02.20	goat	China: Hebei, Baoding
56	Negative	2021.02.20	sheep	China: Hebei, Baoding
57	Negative	2021.02.20	goat	China: Hebei, Baoding
58	Negative	2021.02.20	goat	China: Hebei, Baoding
59	Negative	2021.02.26	sheep	China: Hebei, Dingzhou
60	Negative	2021.02.26	goat	China: Hebei, Baoding
61	Negative	2021.02.27	sheep	China: Hebei, Zhangjiakou
62	Negative	2021.02.27	sheep	China: Hebei, Zhangjiakou
63	Negative	2021.03.12	goat	China: Hebei, Baoding
64	Negative	2021.03.12	goat	China: Hebei, Baoding

65	Negative	2021.03.12	goat	China: Hebei, Baoding
66	Negative	2021.03.15	sheep	China: Hebei, Baoding
67	Negative	2021.03.15	goat	China: Hebei, Baoding
68	Negative	2021.03.15	goat	China: Hebei, Baoding
69	Negative	2021.03.16	sheep	China: Hebei, Dingzhou
70	Negative	2021.03.16	sheep	China: Hebei, Dingzhou
71	Negative	2021.03.16	sheep	China: Hebei, Dingzhou
72	Negative	2021.03.16	sheep	China: Hebei, Dingzhou
73	Negative	2021.03.17	goat	China: Hebei, Baoding
74	Negative	2021.03.17	goat	China: Hebei, Baoding
75	Negative	2021.03.18	sheep	China: Hebei, Baoding
76	Negative	2021.03.18	sheep	China: Hebei, Baoding
77	Negative	2021.03.18	sheep	China: Hebei, Baoding
78	Negative	2022.03.27	goat	China: Hebei, Dingzhou

[a] Each sample was from a different sheep or goat.
